# How do similarities in spatial distributions and interspecific associations affect the coexistence of *Quercus* species in the Baotianman National Nature Reserve, Henan, China

**DOI:** 10.1002/ece3.3863

**Published:** 2018-02-05

**Authors:** Zhiliang Yuan, Boliang Wei, Yun Chen, Hongru Jia, Qingning Wei, Yongzhong Ye

**Affiliations:** ^1^ Henan Agricultural University Zhengzhou Henan China; ^2^ College of Life Sciences Zhejiang University Hangzhou Zhejiang China; ^3^ Educational Administration Department Henan College of Finance and Taxation Zhengzhou Henan China; ^4^ Henan Coal Health School Pingdingshan Henan China

**Keywords:** coexistence, competition, congeneric species, interspecific association, niche theory, spatial pattern

## Abstract

Congeneric species often have similar ecological characteristics and use similar resources. These similarities may make it easier for them to co‐occur in a similar habitat but may also lead to strong competitions that limit their coexistence. Hence, how do similarities in congeneric species affect their coexistence exactly? This study mainly used spatial point pattern analysis in two 1 hm^2^ plots in the Baotianman National Nature Reserve, Henan, China, to compare the similarities in spatial distributions and interspecific associations of *Quercus* species. Results revealed that *Quercus* species were all aggregated under the complete spatial randomness null model, and aggregations were weaker under the heterogeneous Poisson process null model in each plot. The interspecific associations of *Quercus* species to non‐*Quercus* species were very similar in Plot 1. However, they can be either positive or negative in different plots between the co‐occurring *Quercus* species. The spatial distributions of congeneric species, interspecific associations with non‐*Quercus* species, neighborhood richness around species, and species diversity were all different between the two plots. We found that congeneric species did have some similarities, and the closely related congeneric species can positive or negative associate with each other in different plots. The co‐occurring congeneric species may have different survival strategies in different habitats. On the one hand, competition among congenerics may lead to differentiation in resource utilization. On the other hand, their similar interspecific associations can strengthen their competitive ability and promote local exclusion to noncongeneric species to obtain more living space. Our results provide new knowledge for us to better understand the coexistence mechanisms of species.

## 1 INTRODUCTION

Whether congeneric species can stably coexist or not has long been debated (Losos, 2008; Lovette & Hochachka, 2006; Mooney, Jones, & Agrawal, 2008; Münzbergová, 2005). Niche theory predicts that the species coexist because of differences in their resource requirements, so closely related species are less likely to coexist (Cavender‐Bares, Ackerly, Baum, & Bazzaz, 2004; Horner‐Devine & Bohannan, 2006; Slingsby & Verboom, 2006). However, in many temperate forests, species of similar ecological characteristics, for example, congenerics, often coexist at local scales (Davies, Palmiotto, Ashton, Lee, & Lafrankie,^1998^; Yamada, Ngakan, & Suzuki, 2005). Thus, how can these similar congenerics coexist?

Many studies suggest that the similarities in congeneric species are detrimental to their coexistence (Helmus, Savage, Diebel, & Ives, 2007; Sato, Alba, & Sabelis, 2014). The intensity of competition between these species increases with phylogenetic relatedness (Burns & Strauss, 2011; Violle, Nemergut, Pu, & Jiang, 2011). However, many studies have found that the competition between co‐occurring congeneric species is not that strong (Sedio, Wright, & Dick, 2012; Sfenthourakis, Tzanatos, & Giokas, 2006). Some researches suggest that among congeneric species niche differentiation exists (Paoli, 2006) and that discrepancy in resource utilization weakens the competition (McKane et al., 2002; Tanaka et al., 2008). Although congeneric species have similar ecological characteristics, their coexistence is dominated by a neutral process or influenced by other factors, such as resources richness (Andersen, Arnan, & Sparks, 2013; Zhang et al., 2010).

However, other researches have found that not all similar characteristics are harmful. Similarities in fungi or pollinators are beneficial to the coexistence of congeneric species. They can establish symbiotic associations with the same fungi, and the benefit from mycorrhizal networks can effectively alleviate the competition between congeneric species (Dickie, Koide, & Steiner, 2002; Shefferson et al., 2010). In addition, given that some congeneric species share the same pollinators, their coexistence is conducive to their development (Moeller, 2004; Sargent & Ackerly, 2008). Therefore, some similar characteristics of congeneric species can lead to competition, whereas others can lead to mutualism, which helps congeneric species to coexist.

Studies on spatial pattern of congeneric species can compare their similarities and reveal their coexistence mechanisms (Queenborough, Burslem, Garwood, & Valencia, 2007). Although studies on the spatial pattern of congeneric species are available (Zhang et al., 2010), only few examined the similarities in spatial distributions and interspecific associations of congeneric species. Moreover, how these similarities influence the coexistence of congeneric species also remains unknown.

To fill this gap, this study used point pattern analysis to analyze and compare the similarity of spatial patterns, spatial distributions, and interspecific associations of *Quercus* species (*Quercus serrata* var. *brevipetiolata* [QS], *Q. variabilis* [QV], and *Q. aliena* var. *acutiserrata* [QA]) in two 1 hm^2^ plots at the Baotianman National Nature Reserve, Henan, China. Through this study, we hope to answer the following questions: (1) Whether the spatial distributions and spatial patterns of *Quercus* species are similar or not, (2) whether competitions between *Quercus* species are strong or not, and (3) whether interspecific associations of each *Quercus* species with the same non‐*Quercus* species are similar or not.

## 2 METHODS

### 2.1 Study sites and research objects

The Baotianman National Nature Reserve is located in southwest Henan Province in China from 111°46′55″ to 112°03′32″E and 33°35′43″ to 33°20′12″N. The total area is 23,198 ha, average annual temperature is 15.1°C, average annual rainfall is 885.6 mm, average annual evaporation is 991.6 mm, and relative humidity is 68%. The Baotianman National Nature Reserve is located at the transitional area from warm temperate to north subtropical climates (Jia, Chen, Yuan, Ye, & Huang, 2015). Vegetation transitions are from warm temperate deciduous broadleaf forest to subtropical evergreen broadleaf forest. The community mainly consists of natural *Quercus* forest. Among *Quercus* species, QV is mainly distributed below 1,200 m in elevation, QS is mainly distributed between 1,100 and 1,300 m, and QA is mainly distributed above 1,300 m.

**Figure 1:**
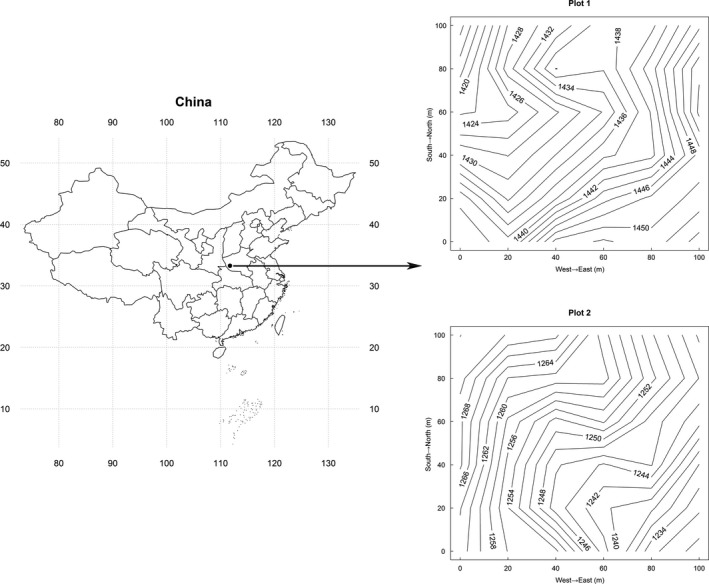
Location and contour maps of the two 1 hm^2^ plots at Baotianman, Henan, China. The number in the contour map is elevation (m), and the unit of (*x*,* y*) axes is meters

**Figure 2:**
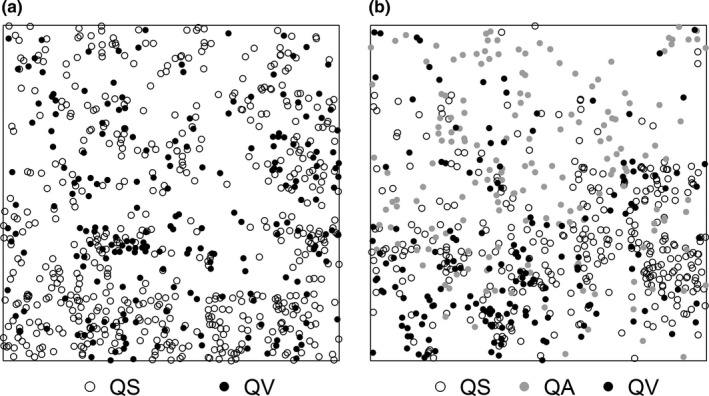
*Quercus serrata* var. *brevipetiolata* (QS) (open circles), *Q. aliena* var. *acutiserrata* (QA) (filled gray circles), and *Q. variabilis* (QV) (filled black circles) in Plot 1 (a) and Plot 2 (b)

The research was carried out in two permanent 1 hm^2^ plots (Figure 1). All living and dead trees with diameters at breast height ≥1 cm were stem mapped, and all individuals were identified to species (Wang et al., 2010). The average elevations of Plots 1 and 2 are 1,271.5 and 1,305.2 m, respectively. The two plots are just at the edge of the main distribution areas of QS, QV, and QA. *Quercus* species were the most dominant species in both plots. Plot 1 had 543 QS stems and 214 QV stems, and Plot 2 had 340 QS stems, 169 QV stems, and 170 QA stems (Figure 2).

As congeneric species, *Quercus* species have some common characteristics. They are all tall deciduous trees and flowering in April to May. Their fruits are nuts, and most of these heavy fruits fall down around the mother tree after maturity. At the same time, some differences in morphological characteristics and life history are found among *Quercus* species (Flora of China Editorial Committee 1998). The cup and size of the acorns, the time for the acorns to mature, and the shapes of leaves are also different. These characteristics may cause divergence in the dispersal of seeds and in the way they use sunlight.

### 2.2 Spatial pattern analysis and null models

#### 2.2.1 Analysis 1: Spatial patterns and spatial distributions

Congeneric species have a common evolutionary history and similar growth habits and responses to habitat (Blomberg & Garland, 2002; Šimková, Ondračková, Gelnar, & Morand, 2002). Therefore, spatial patterns of congeneric species should be similar and show the same pattern in the same habitat. This study used the pair correlation function *g*(*r*) (Equation (1)) (Stoyan & Stoyan, 1994; Wiegand & Moloney, 2004) under the complete spatial randomness null model (CSR) and heterogeneous Poisson's process null model (HP) to analyze the spatial patterns of congeneric species at different scales *r* (Wiegand & Moloney, 2004). In both null models, the position of each point is independent of the position of any other point. In CSR, any point has an equal probability of occurring at any position in the study region. In HP, the points are distributed in accordance with an intensity function λ(*x*,* y*) that varies with location (*x*,* y*) (Wiegand & Moloney, 2004; Zhu, Getzin, Wiegand, Ren, & Ma, 2013; Hypotheses 1, Table 1). *g* (*r*) is related to the derivative of the *K* function (Equation (2)):

(1) $$g\left(r\right)=\frac{dK(r)}{2\pi r\times dr}$$

(2) $$K\left(r\right)=\frac{A}{n^{2}}\sum_{i=1}^{n}\sum_{j=1}^{n}\frac{I_{r}\left(d_{\mathrm{ij}}\right)}{w_{\mathrm{ij}}}\left(i\neq j\right)$$

*A* is the area of the study region, *n* is the number of the points of species, *d*_*ij*_ is the distance between focus point *i* and the other point *j*,* I*_*r*_ is a counter variable (*I*_*r*_(*d*_*ij*_) = 1 if *d*_*ij*_ < *r*, and *I*_*r*_(*d*_*ij*_) = 0 otherwise), and *w*_*ij*_ is a weighting factor to correct for the edge effects (Silva et al., 2016; Wiegand & Moloney, 2004).

**Table 1:** Main hypotheses, applied point pattern analyses, and related figures and tables used in this study

Hypotheses	Point pattern analyses and null models	Related figures and tables
(1) The spatial patterns and spatial distributions of congeneric species are similar	CSR and HP with *g*(*r*) function and RL within a case–control design with the *g*_21_(*r*) − *g*_22_(*r*) using species 1 as the control and species 2 as the case and with *g*′_21_(*r*) − *g*′_22_(*r*) using species 2 as the control and species 1 as the case	Figures 3, 4, 5, 6
(2) Interspecific competitions between congeneric species are weak	HP with bivariate *g*_12_(*r*) function	Figure 7
(3) The interspecific correlations of each congeneric species with the noncongeneric species are similar on the same scales	RL with *g*_13_(*r*) − *g*_23_(*r*): 1 = one congeneric species, 2 = one of the other congeneric species, 3 = one of the noncongeneric species	Tables 2, 3, 4

CSR, complete spatial randomness null model; HP, heterogeneous Poisson's process null model; RL, random labeling null model.

In addition, this study used the bivariate pair correlation function *g*_12_(*r*) (Ripley, 1976, 1977; Stoyan & Stoyan, 1994) under the random labeling null model (RL) and case–control design to test the similarity of spatial distributions between congeneric species in the plots (Getzin, Wiegand, Wiegand, & He, 2008; Zhu et al., 2013). We used species 1 as Pattern 1 and species 2 as Pattern 2. If the results are consistent with RL, then *g*_12_(*r*) = *g*_11_(*r*). This finding indicates that Patterns 1 and 2 have similar spatial distributions in the plots. If individuals of Pattern 2 are relatively more frequent around Pattern 1 than individuals of Pattern 1 around Pattern 1, that is, Pattern 2 shows additional aggregation that is independent from Pattern 1, then *g*_12_(*r*) − *g*_11_(*r*) > 0 (Getzin et al., 2008; Zhu, Mi, Ren, & Ma, 2010). As different species have different spatial distributions, *g*_12_(*r*) − *g*_11_(*r*) and *g*_21_(*r*) − *g*_22_(*r*) should be examined (Hypotheses 1, Table 1).

Consequently, we anticipated three possible results: (1) *g*_12_(*r*) − *g*_11_(*r*) = 0, *g*_21_(*r*) − *g*_22_(*r*) = 0; (2) *g*_12_(*r*) − *g*_11_(*r*) ≠ 0, *g*_21_(*r*) − *g*_22_(*r*) ≠ 0; and (3) *g*_12_(*r*) − *g*_11_(*r*) = 0, *g*_21_(*r*) − *g*_22_(*r*) ≠ 0, or *g*_12_(*r*) − *g*_11_(*r*) ≠ 0, *g*_21_(*r*) − *g*_22_(*r*) = 0. Result 1 shows that the spatial distributions of species 1 and 2 are similar to each other, which indicates that these species have similar response to habitat. Result 2 shows that the spatial distributions of species 1 and 2 are different, indicating that these species may have different response to habitat. *g*_12_(*r*) − *g*_11_(*r*) = 0, *g*_21_(*r*) − *g*_22_(*r*) ≠ 0 in result 3 shows that the spatial distribution of species 1 is similar to species 2, but that of species 2 is quite different from species 1. That is to say, the spatial distribution of species 1 obeys that of species 2. This finding indicates that the response of species 1 to habitat is partly similar to or may be the same as species 2. In the same way, *g*_12_(*r*) − *g*_11_(*r*) ≠ 0, *g*_21_(*r*) − *g*_22_(*r*) = 0 indicates that the response of species 2 to habitat is partly similar to or the same as species 1.

#### 2.2.2 Analysis 2: Interspecific associations

Although similar resource utilization may lead to competitive exclusion of congeneric species, no strong interspecific competition exists between congeneric species in most cases (Queenborough et al., 2007; Valiente‐Banuet & Verdú, 2012; Zhang et al., 2010). As a result, closely related congeneric species are able to coexist (Valiente‐Banuet & Verdú, 2008). To study the interspecific associations in our plots, we fixed the locations of species 1 and used HP to randomize the locations of the individuals of species 2 with the intensity function λ_2_(*x*,* y*), which was based on species 2 (Wiegand & Moloney, 2004). Subsequently, we used bivariate pair correlation function *g*_21_(*r*) to calculate the interspecific association. As the interspecific associations might be asymmetric, we had to examine the *g*(*r*) value in both conditions: species 1 versus species 2 (*g*_12_(*r*)) and species 2 versus species 1 (*g*_21_(*r*)) (Getzin et al., 2006; Wiegand, Gunatilleke, & Gunatilleke, 2007; Hypotheses 2, Table 1).

Given the similar ecological characteristics of congeneric species, the interspecific associations of each congeneric species with the same noncongeneric species may be similar also. That is to say, each of these congeneric species can show negative association with the same noncongeneric species. However, the above method can only detect whether the overall interspecific associations of each congeneric species with noncongeneric species are similar or not. It cannot directly compare whether their interspecific associations at each scale are similar or not. Thus, we used RL as the null model and then fixed *n*_1_ + *n*_2_ locations of species 1 and 2 (representative of two congeneric species). We randomly chose *n*_1_ from these locations as species 1 and the rest of these locations as species 2 (Zhu et al., 2013), and vice versa. Subsequently, we used HP to study the interspecific associations between species 1 (or 2) and 3 (representative of a noncongeneric species). By comparing *g*_13_(*r*) − *g*_23_(*r*) and *g*_31_(*r*) − *g*_32_(*r*), we can directly compare the differences in interspecific associations of different congeneric species with the same noncongeneric species at each scale (Getzin et al., 2006, 2008; Zhu et al., 2013). *g*_13_(*r*) − *g*_23_(*r*) reflects the differences of interspecific associations between species 1 versus species 3 and species 2 versus species 3. If the interspecific associations are similar, then *g*_13_(*r*) − *g*_23_(*r*) = 0; if species 1 has a stronger negative (or positive) influence on species 3 than species 2, then *g*_13_(*r*) − *g*_23_(*r*) < 0 (or *g*_13_(*r*) − *g*_23_(*r*) > 0). Similar to *g*_13_(*r*) − *g*_23_(*r*), *g*_31_(*r*) − *g*_32_(*r*) reflects the differences of interspecific associations between species 3 versus species 1 and species 3 versus species 2 (Hypotheses 3, Table 1).

For all the calculations, we performed edge corrections using Ripley's isotropic edge correction. Details can be found in equation (15.18), page 285, of Stoyan and Stoyan (1994) isotropic edge correction. For all analyses, significant departure from null models on certain scales was evaluated using the lowest and highest value of 199 Monte Carlo simulations to generate approximately 99% simulation envelopes. For the spatial pattern, an observed *g*(*r*) that is higher or lower than the envelope indicates an aggregation or a regular pattern at scale *r*. An observed *g*(*r*) within the envelope indicates a random pattern at scale *r*. For the interspecific association, an observed *g*_21_(*r*) that is higher or lower than the envelope indicates a positive or negative association at scale *r*. An observed *g*(*r*) within the envelope indicates no association at scale *r*. To avoid inflated significance values due to multiple tests across values of *r*, we combined the common simulation envelope method with a goodness‐of‐fit test to assess significant departures from the null model (Diggle, 2003; Loosmore & Ford, 2006). The *p*‐value of the observed pattern is calculated as follows (Equation (3)):

(3) $$\hat{p}=1-\frac{\sum_{i=1}^{s}I\left(u_{0}>u_{i}\right)}{s+1}$$

*u*_*i*_ is a summary statistic that represents the total squared deviation between the observed pattern and the expected result over a distance interval of interest (Wiegand et al., 2007; Zhu et al., 2010). The *u*_*i*_ values were calculated for the observed data (*i *=* *0) and for the data created by the *i *=* *1, … *s* simulations of the null model, where *I*(*u*_0_ > *u*_*i*_) is an indicator function equal to 1 if *u*_0_ > *u*_*i*_ and 0 otherwise. We further analyzed only those data sets with an observed *p*‐value > .05 and a rank >190 (Loosmore & Ford, 2006; Wiegand et al., 2007). All point pattern analyses were conducted using the “spatstat” package in R 2.15.2 (Baddeley & Turner, 2005).

#### 2.2.3 Analysis 3: Neighborhood analysis and species diversity

To detect how the abundant *Quercus* species affect the neighborhood richness in the two plots, an individual‐based approach was used. We determined the relationship between the total basal area of focal species and average species number (or average individual number) in the neighborhood of focal species (Zhang et al., 2009). For each individual of all species in the plot, neighborhoods with radius of 5 m from the focal tree were defined, and the values for each factor were calculated. Simple linear regressions were used to test the relationship among the total basal area and the above‐mentioned factors (Peters, 2003). The Pearson correlation coefficient was used to assess the approximate relationship between the two variables (Benesty, Chen, Huang, & Cohen, 2009; Mukaka, 2012).

Given that different communities may have different diversities, we used Shannon (Equation (4)) (Wagner, Wildi, & Ewald, 2000) and Simpson indices (Equation (5)) (Lexerød & Eid, 2006) to measure the species diversity in each plot:

(4) $$H=-\sum_{i=1}^{n}p_{i}\times \ln p_{i}$$

(5) $$D=1-\sum_{i=1}^{n}p_{i}^{2}$$

*p*_*i*_ is the proportion of basal area of the *i*th species in a quadrat (*p*_*i*_ = *N*_*i*_ */N*), and *N*_*i*_ is the basal area of the *i*th species in a quadrat. *N* is the sum of basal area of all species in the same quadrat.

## 3 RESULTS

### 3.1 Spatial patterns and similarities of congeneric species

**Figure 3:**
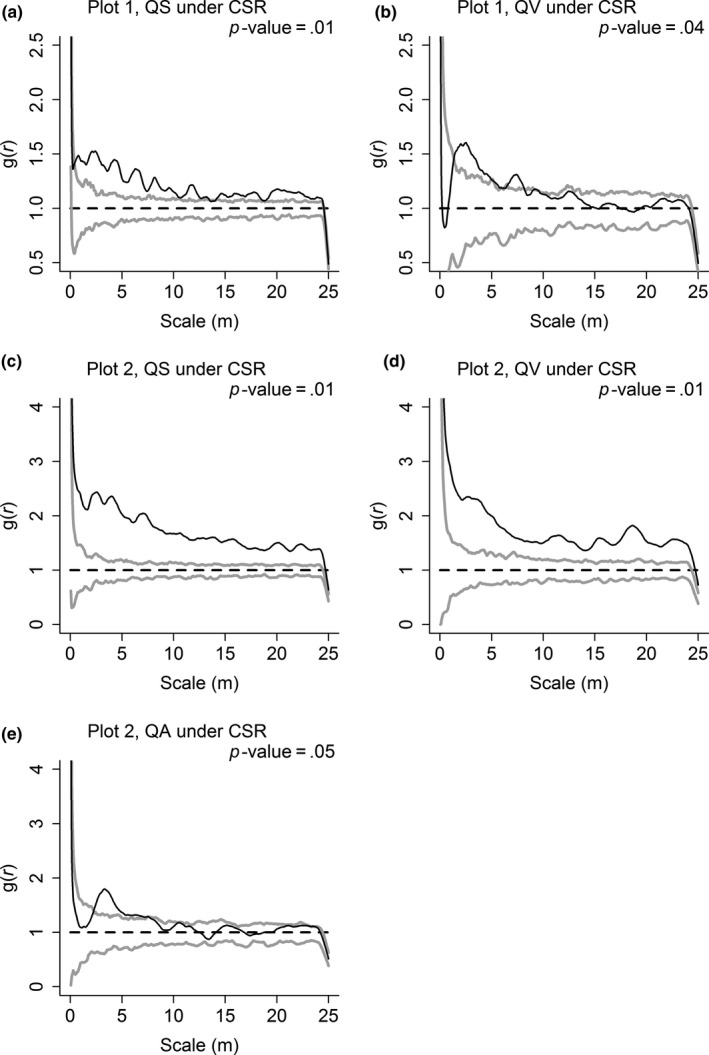
Spatial patterns of *Quercus serrata* var. *brevipetiolata* (QS), *Q. variabilis* (QV), and *Q. aliena* var. *acutiserrata* (QA) in Plot 1 (a and b) and Plot 2 (c, d, and e). The spatial patterns of *Quercus* species are contrasted with complete spatial randomness null model (CSR) using the *g*(*r*) function. Approximately 99% simulation envelopes (gray solid lines) are obtained from 199 Monte Carlo simulations of CSR. The black solid line indicates the observed value, and the black dashed line indicates the theoretical value

**Figure 4:**
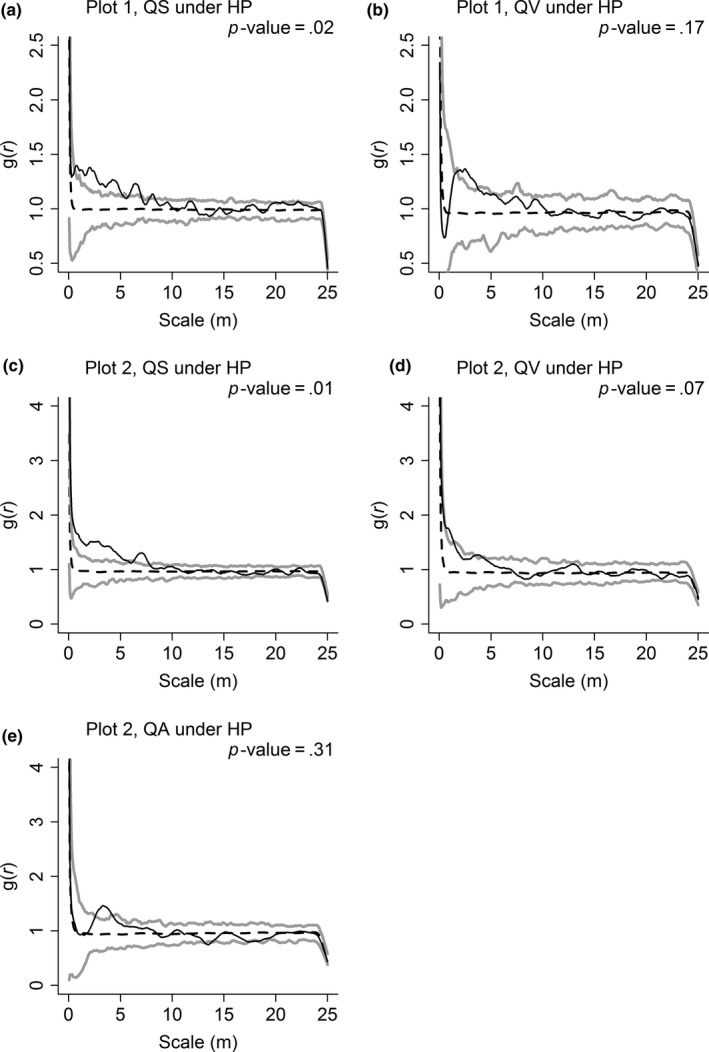
Spatial patterns of *Quercus serrata* var. *brevipetiolata* (QS), *Q. variabilis* (QV), and *Q. aliena* var. *acutiserrata* (QA) in Plot 1 (a and b) and Plot 2 (c, d, and e). The spatial patterns of *Quercus* species are contrasted with heterogeneous Poisson's process null model (HP) using the *g*(*r*) function. Approximately 99% simulation envelopes (gray solid lines) are obtained from 199 Monte Carlo simulations of HP. The black solid line indicates the observed value, and the black dashed line indicates the theoretical value

The spatial patterns of congeneric species were similar in both plots, which all showed significant aggregation under CSR (Figure 3). However, the aggregation was much weaker under HP than CSR. Only QS showed significant aggregation on much fewer scales, and significant aggregation on the large scales (>10 m) under CSR turned random under HP (Figure 4).

**Figure 5:**
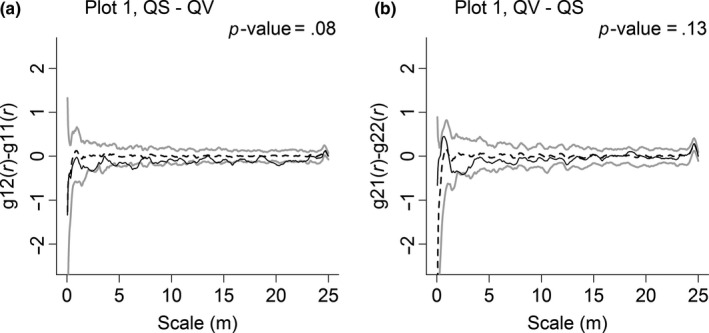
Similarity in spatial distributions of *Quercus serrata* var. *brevipetiolata* (QS) and *Q. variabilis* (QV) in Plot 1. To generate 99% confidence envelopes (gray solid lines), 199 Monte Carlo simulations are used. The black solid line indicates the observed value, and the black dashed line indicates the theoretical value

**Figure 6:**
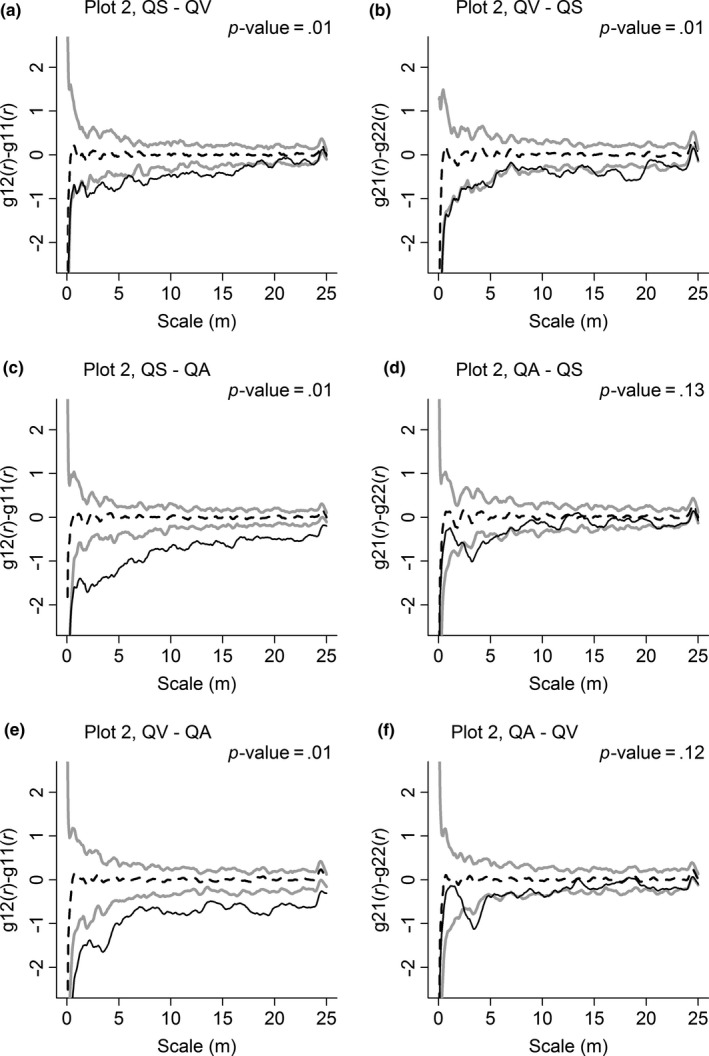
Similarity in spatial distributions of *Quercus serrata* var. *brevipetiolata* (QS), *Q. variabilis* (QV), and *Q. aliena* var. *acutiserrata* (QA) in Plot 2. To generate 99% confidence envelopes (gray solid lines), 199 Monte Carlo simulations are used. The black solid line indicates the observed value, and the black dashed line indicates the theoretical value

The similarities in spatial distributions of congeneric species were different between plots. In Plot 1 (Figure 5), the spatial distributions of QS and QV were quite similar to each other, and their *p*‐values were both higher than .05, which means no significant difference was observed in the spatial distribution between congeneric species. However, in Plot 2 (Figure 6), the spatial distribution of one congeneric species was often different from that of other congeneric species. The spatial distributions of QS and QV were dissimilar to each other and were quite different from those of QA. However, the spatial distribution of QA was similar to that of QS and QV.

### 3.2 Interspecific associations of congeneric species

**Figure 7:**
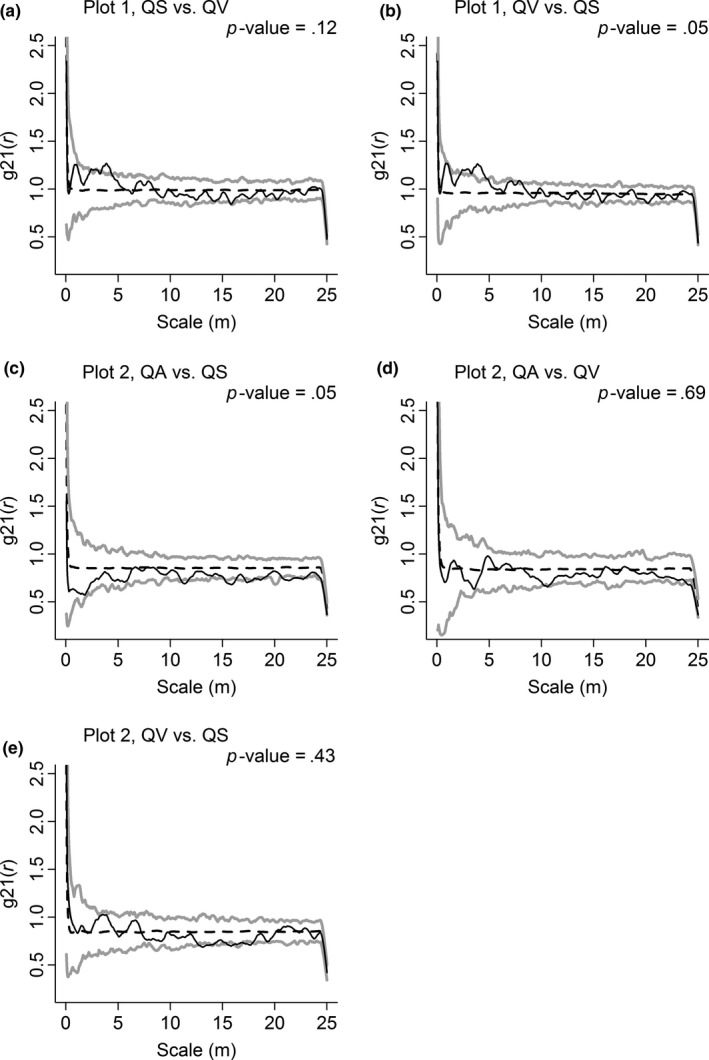
Interspecific associations of *Quercus serrata* var. *brevipetiolata* (QS), *Q. variabilis* (QV), and *Q. aliena* var. *acutiserrata* (QA) in Plot 1 (a and b) and in Plot 2 (c, d, and e). The *g*_12_(*r*) and heterogeneous Poisson's process null model are used to calculate interspecific associations of *Quercus* species. To generate 99% confidence envelopes (gray solid lines), 199 Monte Carlo simulations are used. The black solid line indicates the observed value, and the black dashed line indicates the theoretical value

The interspecific associations among co‐occurring congeneric species were different between the two plots. In Plot 1, QV showed significant positive association with QS, whereas QS showed nonsignificant positive association with QV (Figure 7a and b). In Plot 2, only QA showed a significant negative association with QS. Although the association from QA to QV and from QV to QS was negative in a few scales, their *p*‐values were higher than .05. (Figure 7c,d, and f). QS and QV showed no significant association with QA, and QS showed insignificant association with QV.

Interspecific associations of each congeneric species showed some similarities in Plot 1 (Table 2). Both *Quercus* species showed negative associations with eight of the other 10 species and no association with two species. However, they showed significant negative associations only with four species. Interspecific associations were asymmetrical. Eight of the other 10 species had no significant influences to the *Quercus* species. Only *Sorbus alnifolia* showed significant negative association to both *Quercus* species. *Pyrus calleryana* showed significant negative association to QS.

**Table 2:** Analyses of the spatial associations of each *Quercus* species with non‐*Quercus* species in Plot 1

Species	The influence of *Quercus* species to others				The influence of others to *Quercus* species			
	QS		QV		QS		QV	
*Pyrus calleryana*	−	.01	−	.05	−	.03	−	.24
*Rhododendron simsii*	−	.12	−	.04	+	.28	−	.11
*Forsythia suspensa*	−	.07	−	.29	−	.13	*r*	.42
*Rhododendron mariesii*	−	.01	−	.01	−	.07	−	.12
*Spiraea dasyantha*	−	.06	−	.63	−	.16	−	.85
*Swida walteri*	*r*	.25	*r*	.14	−	.36	*r*	.10
*Fraxinus paxiana*	*r*	.35	*r*	.28	−	.28	*r*	.31
*Sorbus alnifolia*	−	.01	−	.02	−	.04	−	.05
*Dendrobenthamia japonica* var. *chinensis*	−	.02	−	.19	+	.18	*r*	.55
*Pinus tabuliformis*	−	.03	−	.05	−	.15	−	.09

The bivariate statistic of the pair correlation function was used to analyze interspecific associations of each *Quercus* species with non‐*Quercus* species under the heterogeneous Poison's null model. QS means *Q*.* serrata* var. *brevipetiolata*, and QV means *Q*.* variabilis*. “−” and “+” mean the interspecific associations was significant departure from the 99% simulation envelopes which were obtained from 199 Monte Carlo simulations and indicate negative and positive association between species, respectively. “*r*” indicates no association between species. The values in the table are *p*‐values which were calculated from a goodness‐of‐fit test.

However, interspecific associations of each congeneric species were different in Plot 2 (Table 3). Although all three *Quercus* species showed negative associations with seven of the other 10 species, only a few associations were significant: QS showed significant negative association with *Bothrocaryum controversum* and *Sorbus folgneri*, QV showed significant negative association with *Forsythia suspensa* and significant positive association with *Spiraea dasyantha*, and QA showed significant negative association with *B. controversum*. At the same time, six of the other 10 species showed significant negative associations with QS, two species showed significant associations with QV, and no species showed significant associations with QA.

**Table 3:** Analyses of the spatial associations of each *Quercus* species with non‐*Quercus* species in Plot 2

Species	The influence of *Quercus* species to others						The influence of others to *Quercus* species					
	QS		QV		QA		QS		QV		QA	
*Carpinus henryana* var. *henryana*	−	.14	*r*	.50	*r*	.13	−	.04	*r*	.49	*r*	.15
*Bothrocaryum controversum*	−	.03	−	.10	−	.05	−	.03	−	.49	*r*	.16
*Forsythia suspensa*	−	.16	−	.04	−	.42	−	.04	−	.05	*r*	.53
*Spiraea dasyantha*	−	.15	+	.03	*r*	.72	−	.10	+	.02	*r*	.80
*Cornus walteri*	−	.07	−	.67	−	.14	−	.04	−	.62	−	.11
*Castanea seguinii*	*r*	.06	*r*	.16	−	.55	−	.02	−	.12	*r*	.51
*Lindera obtusiloba*	−	.09	−	.15	−	.90	−	.06	−	.15	*r*	.71
*Sorbus folgneri*	−	.05	−	.30	−	.22	−	.29	*r*	.92	−	.52
*Dendrobenthamia japonica* var. *chinensis*	−	.06	−	.19	−	.35	−	.01	−	.08	−	.26
*Euonymus alatus*	−	.18	−	.07	−	.46	−	.26	−	.16	*r*	.81

The bivariate statistic of the pair correlation function was used to analyze interspecific associations of each *Quercus* species with non‐*Quercus* species under the heterogeneous Poison null model. QS means *Q*.* serrata* var. *brevipetiolata*, QV means *Q*.* variabilis*, and QA means *Q*. *aliena* var. *acutiserrata*. “−” and “+” mean the interspecific associations significantly departure from the 99% simulation envelopes which were obtained from 199 Monte Carlo simulations and indicate negative and positive association between species, respectively. “*r*” indicates no association between species. The values in the table are *p*‐values which were calculated from a goodness‐of‐fit test.

### 3.3 Similarities in interspecific associations of each *Quercus* species

In Plot 1, the interspecific associations of QS and of QV were similar with the same non‐*Quercus* species at each scale. The interspecific associations of QS and of QV were significantly different only with *P. calleryana*. The interspecific associations of the other 10 species to QS and to QV all showed no significant differences.

In Plot 2, however, we found that the interspecific associations of each *Quercus* species were not exactly the same on each scale. The interspecific associations of each *Quercus* species showed more differences on each scale in Plot 2 than in Plot 1 (Table 4). In the interspecific associations with non‐*Quercus* species, QS and QV showed significantly different with three species; QS and QV showed significantly different with four species; QS and QV showed significantly different with six species. Meanwhile, the interspecific associations of non‐*Quercus* species to each *Quercus* species also showed some differences at each scale (Table 4).

**Table 4:** Comparisons of interspecific associations of each *Quercus* species with one species in Plot 2

Differences	The influence of *Quercus* species to others			The influence of others to *Quercus* species		
	QS–QV	QS–QA	QA–QV	QS–QV	QS–QA	QA–QV
*Bothrocaryum controversum*	.03*	.01*	.01*	.01*	.01*	.01*
*Carpinus henryana* var. *henryana*	.74	.16	.04*	.69	.31	.46
*Castanea seguinii*	.03*	.01*	.12	.09	.01*	.19
*Dendrobenthamia japonica* var. *chinensis*	.13	.02*	.01*	.10	.04*	.03*
*Euonymus alatus*	.85	.06	.05*	.72	.32	.65
*Forsythia suspensa*	.08	.38	.01*	.27	.44	.05*
*Lindera obtusiloba*	.38	.13	.01*	.35	.14	.01*
*Sorbus folgneri*	.81	.74	.60	.94	.68	.74
*Spiraea dasyantha*	.08	.26	.10	.01*	.66	.04*
*Swida walteri*	.03*	.01*	.15	.11	.05*	.34

QS means *Q*. *serrata* var. *brevipetiolata*, QV means *Q*. *variabilis*, and QA means *Q*.* aliena* var. *acutiserrata*. The values in the table are *p*‐values. “*” means the *p*‐value of result was larger than .05, which means there were significant difference in interspecific association of each *Quercus* species to a non‐*Quercus* species.

### 3.4 Neighborhood competition and species diversity

**Figure 8:**
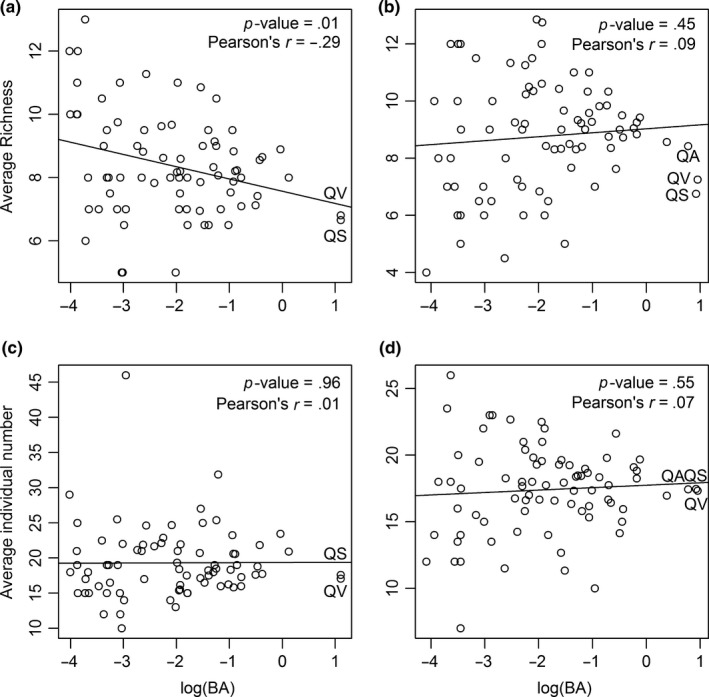
Neighborhood analysis of all species in Plot 1 (a and c) and in Plot 2 (b and d). (a and b) Are the relationships between the number of neighborhood species and the total basal area of focal species; (c and d) are the relationships between the number of neighborhood individuals and the total basal area of focal species

In both plots, the *Quercus* species were all abundant. They all had less neighborhood richness around them. However, only in Plot 1 was the number of neighborhood species significantly negatively correlated with the total basal area of focal species, and the number of neighborhood individuals had no correlation with the total basal area (Figure 8a and c). In Plot 2, neither the number of neighborhood species nor the number of neighborhood individuals was correlated with the total basal area of the focal species (Figure 8b and d). At the same time, Plot 1 had lower diversity than Plot 2: the Shannon index was 1.55 in Plot 1 and 2.13 in Plot 2; the Simpson index was 0.66 in Plot 1 and 0.81 in Plot 2.

## 4 DISCUSSION

The congeneric species evolve from the same ancestor, and most of them have close phylogenetic relationships and therefore have similar characteristics in many aspects (Lavorel & Garnier, 2002; Wiens et al., 2010). The closely related *Quercus* species all showed aggregation spatial patterns under CSR, and the aggregations were weaker under HP in both plots. This finding indicates that the distributions of *Quercus* species were more consistent with HP than with CSR, that is, the distributions were more or less affected by the environment.

At the same time, the spatial distribution of *Quercus* species in Plot 1 showed no significant difference (Figure 5), suggesting similar resource utilization. The similarities in the interspecific associations of congeneric species were discovered for the first time. The *Quercus* species always showed either negative or no influence on the same non‐*Quercus* species, and the interspecific associations of each *Quercus* species to the non‐*Quercus* species were always similar on each scale in Plot 1. This finding indicates that congeneric species not only have similar responses to the habitat but also are likely to interact with the same species in the community (Zhang et al., 2010).

However, the similarities of these characteristics can be changed through different plots: Spatial distributions of co‐occurring *Quercus* species were different in Plot 2 (Figure 6), suggesting differentiation in resource utilization. Additionally, the significant interspecific associations of each *Quercus* species to the same non‐*Quercus* species were different (Table 3). Moreover, interspecific associations of each *Quercus* species to the same non‐*Quercus* species on each scale were more different in Plot 2 than that in Plot 1 (Table 4). These findings indicate that congeneric species may have different responses to the habitat, and interspecific associations with the same non‐*Quercus* species may vary from *Quercus* species.

Strangely, similarities in congeneric species did not necessarily cause strong competition among them. Co‐occurring *Quercus* species showed more similarities in Plot 1 than in Plot 2 (Table 5). However, the *Quercus* species were not likely to compete with each other as there were positive association in Plot 1 (Figure 7a and b) but they may have competition as there were negative association in Plot 2 (Figure 7c–e). This phenomenon may be related to other environmental factors, such as soil conditions. Burns and Strauss (2011) also found that close relatives can compete more with each other or might have more mutualistic relationships than distant relatives under different soil conditions. As the spatial patterns of *Quercus* species are affected by the environment, these congeneric species use the same resources without competition when the resources in the soil are abundant (Plot 1). However, when the resources are limited, they have to compete with each other, which may lead to niche differentiation in congeneric species (Plot 2) (Beltrán, Valiente‐Banuet, & Verdú, 2012; Chase, 2003). In addition, when canopy trees are in their early age, asymmetric competition with the overstory may also reduce resource competition, which helps the co‐occurrence of closely related species (Sedio et al., 2012).

**Table 5:** Similarities and differences in the two plots

	Plot 1	Plot 2
(1) Spatial patterns	*Quercus* species showed aggregation patterns under CSR, and the aggregation was weaker under HP than under CSR	
(2) Interspecific associations of congeneric species to noncongeneric species	*Quercus* species showed either negative or no influences on noncongeneric species	*Quercus* species showed few significant associations with noncongeneric species
(3) Interspecific associations among congeneric species	Positive spatial associations with each other	Negative spatial association with each other
(4) Spatial distributions of congeneric species	No significant difference from each other	Quite different from each other
(5) Similarities in interspecific associations of each congeneric species to a noncongeneric species	Similar	Different
(6) Relationships between basal area and neighborhood richness in the community	Significant correlations	No significant correlation

At the same time, negative associations of each *Quercus* species with non‐*Quercus* species were much stronger in Plot 1 than in Plot 2 (Tables 2 and 3), and species diversity in Plot 1 was lower than that in Plot 2. This finding is probably because *Quercus* species did not compete with each other and influenced the same species on the same scales in Plot 1 (Figure 7a and b). These actions may strengthen the competition of congeneric species and help them to combine together to remove noncongeneric species and to obtain more living space (Bengtsson, Fagerström, & Rydin, 1994; Leege, Thompson, & Parris, 2010). Thus, the rare species were more likely to be excluded from the community, so species diversity in Plot 1 was lower than that in Plot 2 (Connell & Slatyer, 1977; Zhang, Duan, Xian, Korpelainen, & Li, 2011). However, as *Quercus* species were negatively associated with each other and non‐*Quercus* species on different scales in Plot 2 (Figure 7c–e; Table 4), they were not able to combine together and influence non‐*Quercus* species on the same scales. Given the weak competition of *Quercus* species, they were not able to exclude noncongeneric species, so species diversity was high in Plot 2. Hence, congeneric species may compete with non‐*Quercus* species to obtain more living space, or they may compete with each other for the limited resource.

*Quercus* species were the most abundant species in terms of basal area, but they had less diverse local communities in Plot 1. However, it was not an artifact of *Quercus* species to have fewer individuals in the neighborhood (Figure 8). Given that basal area was not correlated with the number of individuals, more conspecific individuals may be found around *Quercus* species than heterospecific individuals. Results indicate that *Quercus* species may have strong interspecific competition with non‐*Quercus* species (Figure 8a), but the intraspecific competition of *Quercus* species was weak. However, no relationship between basal area and average richness was found in Plot 2 (Figure 8b), which means that interspecific competition between *Quercus* species and non‐*Quercus* species was not that strong. Obviously, *Quercus* species have different survival strategies under different habitats.

## 5 CONCLUSION

Congeneric species have some similar ecological characteristics such as similar spatial pattern and spatial distribution. We also discovered similarities in the interspecific associations of congeneric species for the first time. Although the similarity in the use of resources may lead to competition, similar influence to noncongeneric species can provide an opportunity for congeneric species to strengthen their competitive ability and promote their coexistence. Thus, to obtain sufficient resources, congenerics may compete with each other until they have enough differentiation in resource utilization, or they may combine together to exclude the noncongeneric species to obtain more living space. However, under different environments, congeneric species may change their survival strategies. Environmental factors and similar interspecific associations can affect the coexistence of congeneric species. However, which factor plays the most important role and in which way do these factors affect the coexistence still need further research.

## CONFLICT OF INTEREST

The authors declare that they have no conflict of interest.

## AUTHOR'S CONTRIBUTIONS

BLW, ZLY, and ZYY conceived the ideas and designed methodology; YC and JHR collected the data; BLW and ZLY analyzed the data; BLW, ZLY, and QNW led the writing of the manuscript. All authors contributed critically to the drafts and gave final approval for publication.

## ETHICAL APPROVAL

This article does not contain any studies with human participants or animals performed by any of the authors.
